# Profiling of high-grade central osteosarcoma and its putative progenitor cells identifies tumourigenic pathways

**DOI:** 10.1038/sj.bjc.6605405

**Published:** 2009-11-03

**Authors:** A-M Cleton-Jansen, J K Anninga, I H Briaire-de Bruijn, S Romeo, J Oosting, R M Egeler, H Gelderblom, A H M Taminiau, P C W Hogendoorn

**Affiliations:** 1 Department of Pathology, Leiden University Medical Center, PO box 9600, Leiden 2300 RC, The Netherlands; 2 Department of Paediatric Immunology, Haematology, Oncology and Bone Marrow Transplantation, Leiden University Medical Center, PO box 9600, Leiden 2300 RC, The Netherlands; 3 Department of Clinical Oncology, Leiden University Medical Center, PO box 9600, Leiden 2300 RC, The Netherlands; 4 Department of Orthopaedic Surgery, Leiden University Medical Center, PO box 9600, Leiden 2300 RC, The Netherlands

**Keywords:** mesenchymal stem cells, osteoblastoma, bone tumour, RNA expression profile, Wnt signalling

## Abstract

**Background:**

Osteosarcoma is the most prevalent primary malignant bone tumour in children and young adults, with poor survival in 40% of patients. To identify the signalling pathways involved in tumourigenesis, we compared gene expression in osteosarcoma with that in its presumed normal counterparts.

**Methods:**

Genome-wide expression profiles were generated from 25 high-grade central osteosarcoma prechemotherapy biopsies, 5 osteoblastomas, 5 mesenchymal stem cell (MSC) populations and these same MSCs differentiated into osteoblasts. Genes that were differentially expressed were analysed in the context of the pathways in which they function using the GenMAPP programme.

**Results:**

MSCs, osteoblasts, osteoblastomas and osteosarcomas clustered separately and thousands of differentially expressed genes were identified. The most significantly altered pathways are involved in cell cycle regulation and DNA replication. Several upstream components of the Wnt signalling pathway are downregulated in osteosarcoma. Two genes involved in degradation of *β*-catenin protein, the key effectors of Wnt signalling, *Axin* and *GSK3*-*β,* show decreased expression, suggesting that Wnt signalling is no longer under the control of regular signals. Comparing benign osteoblastomas with osteosarcomas identified cell cycle regulation as the most prominently changed pathway.

**Conclusion:**

These results show that upregulation of the cell cycle and downregulation of Wnt signalling have an important role in osteosarcoma genesis. Gene expression differences between highly malignant osteosarcoma and benign osteoblastoma involve cell cycle regulation.

Osteosarcoma is the most common primary bone malignancy, with a yearly incidence of approximately 6 per million children and 2 per million adults (Raymond *et al*, 2002). Peak incidence occurs in late puberty, with 50% of patients being between 10 and 20 years of age, and 60% younger than 25 years. Osteosarcoma in patients over 40 years of age is, in a substantial number of cases, generally considered secondary, such as after exposure to irradiation, or it arises in areas of preexisting Paget's disease of bone (Fuchs and Pritchard, 2002). It might thus be considered as a different disease than osteosarcoma in young patients.

Several histological subtypes are distinguished, of which conventional high-grade central or intramedullary osteosarcoma is the most common (75%) (Fletcher *et al*, 2002). The aetiology of high-grade central osteosarcoma in young patients is elusive. No benign or malignant precursor lesions are known. These tumours recapitulate osteogenesis, compliant with their capacity to produce osteoid, alkaline phosphatase, osteocalcin, osterix and bone sialoprotein.

The outcome for patients with high-grade osteosarcoma has improved substantially since the introduction of multimodal chemotherapy, with present overall survival rates ranging from 65 to 75%. However, this improvement has reached a plateau despite several trials opting for intensifying the dose or applying alternative chemotherapy schedules. Increasing the dose of chemotherapy before surgery only improved the response rate, but not survival (George, 2007; Lewis *et al*, 2007b). To treat patients who are refractory to chemotherapy or those who relapse, alternative targets for therapy are required that can be identified through knowledge of molecular biological characteristics of the tumour.

Molecular studies on osteosarcoma are greatly hampered by the enormous genetic instability that obscures the identification of genetic loci involved in osteosarcoma genesis (Hogendoorn *et al*, 2003), and furthermore by the lack of benign precursors and no certainty of the normal counterpart or progenitor cells. Osteoblastoma is a benign bone tumour occurring at the same site, but this tumour has never been reported to progress to osteosarcoma. A potential cell-of-origin of osteosarcomas is the mesenchymal stem cell (MSC), the precursor of osteoblasts as has been suggested in mouse models (Tolar *et al*, 2007). Genome-wide expression profiling to identify genes that are involved in response to chemotherapy and survival of osteosarcoma has been reported (Ochi *et al*, 2004; Man *et al*, 2005; Mintz *et al*, 2005). In all, 104, 44 and 60 differentially expressed genes were reported when comparing good and poor responders to chemotherapy. Remarkably, these lists of genes do not overlap by one single gene.

Here we report on a genome-wide expression profiling study on a homogeneous series of high-grade central osteosarcomas of patients younger than 40 years of age. Using strict criteria to correct for multiple testing, we were unable to identify genes that were significantly different when comparing good and poor responders. A comparison of osteosarcoma expression profiles with the putative progenitor cells of osteosarcoma, that is, MSCs and the same MSCs differentiated into osteoblasts, resulted in the identification of large sets of genes that show very significant differential expression. These genes could be grouped according to signal transduction pathways in which they function, thereby identifying possible culprit molecular events responsible for osteosarcoma genesis.

## Materials and methods

### Patient material and mesenchymal stem cells

Patients and their clinical data are listed in Table 1. All patients were treated at Leiden University Medical Center (LUMC). For osteosarcoma patients, the difference in response to chemotherapy was stratified as good or poor response, using the Huvos criteria (Huvos, 1991). Good response was defined if <10% of tumour cells are viable after pre-operative chemotherapy, poor response if more than 10% of tumour cells are viable. This response rate has been shown to be the best predictive marker for prognosis (Bielack *et al*, 2002). Chemotherapy protocols include both pre- and post-operative treatment and were comparable (Lewis *et al*, 2007a). Osteoblastoma patients were treated with surgery only. The difference in survival of osteosarcoma was stratified as good if patients were still alive after 5 years of follow-up, whereas poor survivors were patients who died from their disease within this time window. The disease course for osteoblastoma patients was usually without remission, except for recurrence in one patient.

Ribonucleic acid was extracted from frozen biopsies that were obtained before pre-operative chemotherapy was administered. For osteosarcoma, core biopsies with at least 70% tumour cells and with non-necrotic tissue were used in this study. For osteoblastoma, resected tumours were used for RNA extraction.

We used human bone-marrow-derived mesenchymal stem cells and osteoblasts derived from the same cells on osteogenic differentiation. Cells were isolated from bone marrow samples as previously described (Bernardo *et al*, 2007b). MSC1, MSC2 and FMSC1 were obtained from the Department of Hematology, Leiden University Medical Center, Leiden, The Netherlands. 220L and 240R were purchased from Tulane University, New Orleans, LA, USA. All cells used were derived from adult patients, except for FMSC1, which was derived from foetal bone marrow, and were obtained according to the ethical guidelines of the national organisation of scientific societies (FEDERA). All cells were characterised either at passage 2 or passage 3 through FACS analysis as previously described (Bernardo *et al*, 2007a). The phenotypes were uniform among all the different cells tested and in agreement with those reported for MSCs, that is, CD90, CD105, CD166, HLA-A, B and C positive (>95%), and CD34, CD 45, CD31, CD80 and HLA-DR negative (<5%). Furthermore, all cells were tested for their ability to be committed under proper conditions towards adipogenesis, chondrogenesis and osteogenesis, as previously described (Bernardo *et al*, 2007a). All cells that were induced to osteogenic differentiation showed a diffused positive staining for alkaline phosphatase activity and alizarin red positive calcium depositions, as previously described (Bernardo *et al*, 2007a).

All tissue samples were handled in a coded manner, according to the National Ethical Guidelines (‘Code for Proper Secondary Use of Human Tissue in The Netherlands’, Dutch Federation of Medical Scientific Societies, http://www.federa.org).

### Expression array analysis

Ribonucleic acid was extracted from frozen tissue sections of 20 *μ*m as described previously (Baelde *et al*, 2001). Generation of cRNA and labelling were performed according to the Affymetrix protocol. Briefly, 10 *μ*g of RNA was used to generate double-stranded cDNA by an oligo-dT primer and a T7-RNA polymerase promotor. Reverse transcription and subsequent amplification and labelling were carried out in accordance with protocols recommended by Affymetrix using the BioArray HighYield RNA Transcript Labeling kit (ENZO Life Sciences, Farmingdale, NY, USA). Every step of the reverse transcription and labelling procedure is monitored by gel electrophoresis and spectrophotometry.

Labelled RNA is hybridised with Hu133A GeneChip Arrays (Affymetrix, Santa Clara, CA, USA) according to the manufacturer's protocol (http://www.affymetrix.com/support/technical/manuals.affx) and scanned on an Affymetrix GeneChip scanner.

The quality of hybridisation is assessed by calculating the ratio of 5′ and 3′ features for the reference genes, GAPDH and actin. When this ratio is greater than 2, it indicates a measure of poor quality and the chip is discarded.

All expression array data are available at the BJC online supplementary material website.

### Data analysis

GeneChip data were normalised using GC-RMA, an algorithm provided by the Bioconductor project (http://www.bioconductor.org/), which considers only perfect match values (Gautier *et al*, 2004). The algorithm runs under statistical language *R* and was shown to give less false-positive variance in technical duplicates and has a greater sensitivity and specificity (Irizarry *et al*, 2003), as was recently confirmed in our laboratory (Sieben *et al*, 2005).

The Spotfire decision site for functional genomics was used to perform unsupervised hierarchical clustering on all genes with a variance of at least 0.5.

To select genes that can be used as classifiers for histological response on preoperative treatment and survival, the Limma (linear models for microarray data) package of Bioconductor (http://www.bioconductor.org) was applied to the data set. Limma is a moderated *T*-statistic that detects differentially expressed genes between groups, given the natural variance within these groups, corrected for the false discovery rate due to multiple testing (Wettenhall and Smyth, 2004).

For pathway analysis, array data were mined with GO-Elite, a tool to identify pathways that are most significantly changed between groups (http://www.genmapp.org/go_elite/go_elite.html and PMID: 15961447). To visualise gene expression data in biological pathways, GenMAPP was used (Dahlquist *et al*, 2002).

Quantitative reverse transcriptase PCR was performed as described previously (Rozeman *et al*, 2005). Primers for control genes and *WNT5A* have been submitted to the Real Time PCR Primer and Probe Database (http://medgen.ugent.be/rtprimerdb/).

## Results

### Comparing expression profiles of osteosarcomas

For 25 pre-operative biopsies from high-grade central osteosarcomas, we obtained good-quality genome-wide expression data. One sample was repeated twice and three were repeated once to test for technical reproducibility. All four samples were most similar to their duplicates as demonstrated by hierarchical clustering, as replicates always clustered together (data not shown). For further analyses, we used only one of the replicates. The entire file containing all expression profiling data can be found in supplementary Table 1.

Hierarchical clustering of all osteosarcoma profiles did not result in separation into groups, implying no big differences between possible clinical subsets. Previous publications reported that there are significantly differentially expressed genes when comparing osteosarcomas from patients with good *vs* poor response to chemotherapy (Man *et al*, 2005; Mintz *et al*, 2005). However, we could not identify any significantly expressed gene when comparing good and poor responders when applying a moderated *T*-statistic that corrects for multiple testing as described in the methods section.

For all patients, at least 5 years of follow-up data were available. Poor survivors are defined as those having less than a 5-year survival period compared with good survivors with more than a 5-year survival period. The same *T*-statistic was used for the classification in good and poor survival; however, no significantly differentially expressed genes were acknowledged and thereby no prognostic markers were identified.

### Genes differentially expressed based on comparison of cultured cells and primary tissue

**Figure 1:**
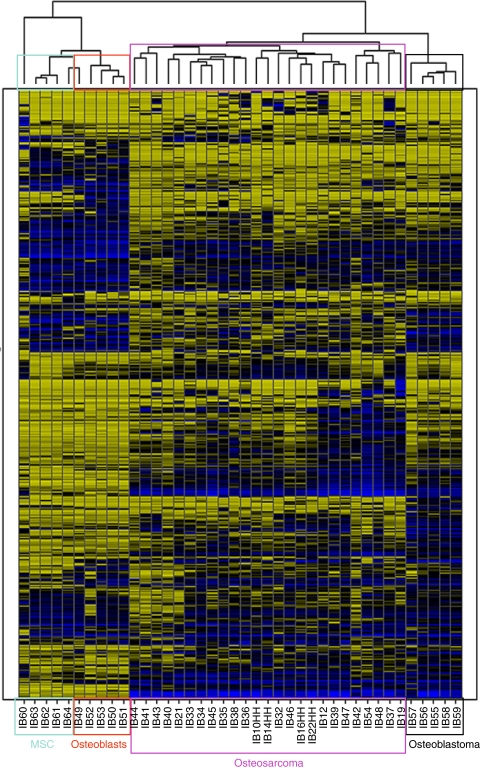
Hierarchical clustering. Hierarchical clustering of expression profiling data clearly shows separate clusters for osteosarcomas, osteoblastomas, mesenchymal stem cells (MSCs) and the same MSCs differentiated into osteoblasts.

To identify the biological processes involved in osteosarcoma genesis, the expression profiles of the 25 osteosarcomas were compared with profiles of the presumed progenitors of this tumour, that is, bone marrow-derived MSCs (*n*=5) and osteoblasts derived from these MSCs (Bernardo *et al*, 2007b). Furthermore, profiles of five osteoblastomas were included that are not considered as benign precursors, as these tumours were never reported to progress to osteosarcoma. Hierarchical clustering clearly distinguished the four groups into separate clusters (Figure 1). The *t*-test in Limma assigned many significantly differentially expressed genes when carrying out pair-wise comparisons (Table 2).

**Figure 2:**
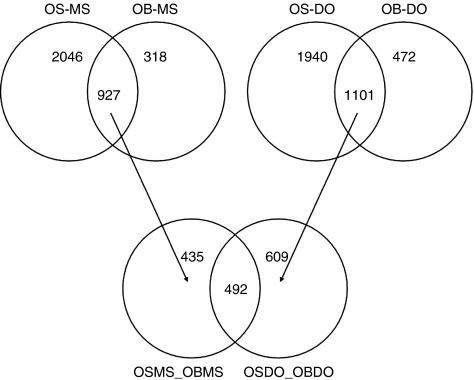
Venn diagram of the ‘culture-tissue’ gene subset. The circles from these VENN diagrams represent the differentially expressed genes when comparing two groups of arrays. The overlap between two circles contains genes that are the same in both comparisons. OS=osteosarcomas; OB=osteoblastomas; MS=mesenchymal stem cells; DO=MSCs differentiated into osteoblasts. The lower VENN diagram shows the overlap of the 492 differentially expressed genes when comparing expression profiles from primary tissue (OS, osteosarcoma and OB, osteoblastoma) with those from cultured cells (MS MSCs and DO, differentiated into osteoblasts). The circle OSMS_OBMS contains all genes differentially expressed when comparing osteosarcoma and MSCs that overlap with the differentially expressed genes when comparing osteoblastoma and MSC. OSDO_DOOB is the same as OSMS_OBMS, but for MSCs differentiated into osteoblasts.

**Figure 3:**
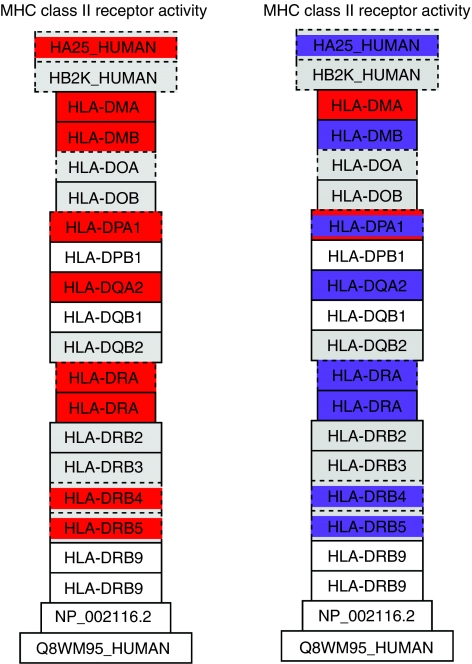
MHC class II normal *vs* tissue culture related. The MHC class II receptor activity pathway with genes that are differentially expressed between osteosarcoma and mesenchymal stem cells (MSCs) coloured. Green is upregulated in osteosarcoma, purple indicates that a gene belongs to the 492 genes of the culture-tissue set. The left panel was analysed without taking this set into account, the right set with the ‘culture-tissue’ gene set as the first parameter. The color reproduction of this figure is available on the html full test version of the manuscript

The GO-Elite programme selected pathways that are most significantly different when comparing groups, namely, GO-Elite ranks pathways with excess of differentially expressed genes. One of the most significant pathways when comparing MSCs with osteosarcoma was the MHC class II receptor activity pathway, which was upregulated in osteosarcoma. It is difficult to understand how the increase in such a pathway could contribute to mesenchymal transformation. We hypothesised that some of the genes identified by the *t*-test are merely different because cultured cells (MSCs) are compared with primary tissue. The genes that are most likely to belong to this category are those that show a similar expression in cultured MSCs and osteoblasts, as well as in primary osteosarcoma and osteoblastoma, but significant differences between the group of cultured cells and primary tissues. To identify these genes, Venn diagrams were made of all differentially expressed genes for all comparisons using the Limma package from Bioconductor (http://www.bioconductor.org). A final Venn diagram (Figure 2) identified 492 genes that are likely to be different because of the comparison of cultured cells with primary tissue. The overlapping category in Figure 2 consists of all genes that are significantly different when cultured cells are compared with tissue, for both the highly malignant osteosarcomas and the benign osteoblastomas. The procedure to construct VENN diagrams is explained in the legend of Figure 2. GennMAPP analysis was performed on the entire data set, with the ‘culture-tissue’ category being marked as the leading parameter in the expression data set marked purple. The group of eight genes in the MHC class II receptor pathway that had a *P*-value of <0.05 seems to consist of seven genes that were assigned to the purple-coloured ‘culture-tissue’ category (Figure 3). This suggests that the approach to filter out genes that may be the result of comparing cultured cells and tissue is a valid one. However, this approach has its limitations because separate genes cannot be validated with a gold standard, nor can they be excluded, as there are genes in this set that are similarly differentially expressed between MSCs *in vivo vs* both osteoblastomas and osteosarcomas.

### Comparing osteosarcoma with its presumed progenitors

**Figure 4:**
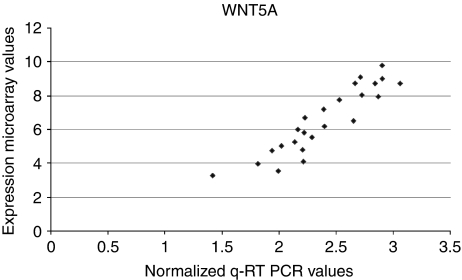
Quantitative RT-PCR for WNT5A. Comparison of q-RT-PCR with array data for WNT5A shows 92% correlation.

The 25 osteosarcomas as a single group compared with five cultures of undifferentiated mesenchymal stem cells. This resulted in a substantial number of 2973 differentially expressed genes (corrected *P*-value<0.05), of which 1159 genes are higher expressed in MSCs than in osteosarcomas and 1814 lower. We further compared osteosarcomas with the same MSC cultures differentiated into osteoblasts. This resulted in 3041 differentially expressed genes (*P*<0.05). Table 2 summarises the results of all comparisons made. There is a large overlap of 1725 genes in osteosarcomas *vs* MSCs and osteosarcomas *vs* differentiated osteoblasts (DO). One gene that was significantly less expressed in osteosarcoma was *WNT5A*. This gene, involved in non-*β*-catenin Wnt signalling (Kuhl *et al*, 2000), has been tested with quantitative RT–PCR on the same series of RNA that has been used on microarrays as an alternative method to verify array data. The correlation between qPCR and array data was good, that is, 92% (Figure 4).

Given the high number of significantly differentially expressed genes, we did not consider it relevant to make a shortlist of the most significant genes. Instead, the programme, GO-Elite, was used to identify pathways with a high number of differentially expressed genes and GENMAPP was used to specifically consider pathways that are known to be involved in normal osteoblast differentiation. For GO-Elite analysis, we removed the 492 ‘culture-tissue’ artefact genes from the significant list.

Table 3 lists pathways that contain most differentially expressed genes when comparing MSCs and osteosarcoma. Pathways in this table have an adjusted *P*-value smaller than 0.05 upon strict statistical criteria, that is, those by Benjamini and Hochberg (1995). The significant pathways are associated with DNA replication and mitosis, of which several genes involved in positive regulation are upregulated in osteosarcoma, such as *CCNB* when compared with MSC. None of the significant genes in these pathways are identified as ‘culture-tissue artefacts’.

**Figure 5:**
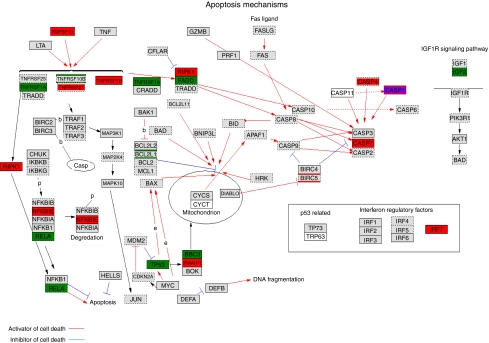
The p53 pathway is upregulated in osteosarcoma. The apoptosis/p53 pathway components when comparing osteosarcoma with differentiated osteoblasts, with genes upregulated in osteosarcoma in green (dark green when the *P*-value <0.01, light green when *P*<0.05) and genes downregulated in red (red, *P*-value<0.01, pink *P*<0.05). The color reproduction of this figure is available on the html full test version of the manuscript

To further mine data, we considered specific pathways that are known or suspected to be involved in osteosarcoma genesis. Inactivation of the p53 pathway has been reported in osteosarcoma (Wunder *et al*, 2005) and this is indeed confirmed when comparing expression profiles from osteosarcoma with its presumed progenitor, MSCs and osteoblasts. Figure 5 shows the p53-mediated apoptotic pathway with genes that are downregulated in osteosarcoma (*P*<0.05) in green. Downregulation of p53-mediated signalling is reflected by downregulation of the specific downstream gene, *BBC3/PUMA*.

**Figure 6:**
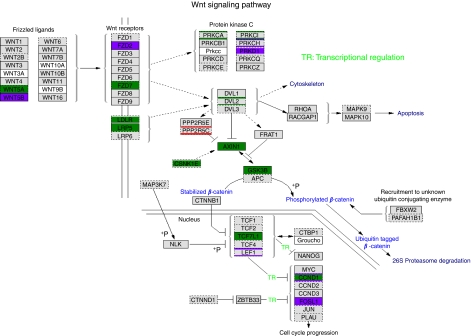
Wnt signalling pathway downregulated in osteosarcoma. The Wnt signalling pathway when comparing osteosarcoma with mesenchymal stem cells (MSCs), legend is the same as Figure 5. The color reproduction of this figure is available on the html full test version of the manuscript

The Wnt pathway has been shown to have an important role in osteoblast differentiation (Hartmann, 2006) and therefore, in this study, we visualised this pathway with GenMAPP application using expression data. Wnt signalling seems downregulated when comparing MSCs or differentiated osteoblasts with osteosarcomas. Figure 6 shows the Wnt pathway when comparing osteosarcomas and MSCs. The picture is similar when comparing with osteoblasts, although less prominent. Both upstream, the Wnt receptors *FZD*2 and -7 and *LRP*5 as downstream *CCND*1 and *AXIN* are downregulated.

### Osteosarcoma *vs* osteoblastoma

Expression profiles of osteosarcoma were compared with those of five osteoblastomas, a benign bone tumour occurring at a similar site in long bones and in a similar age group as osteosarcoma. The large difference in disease course is reflected by a large set of significantly differentially expressed genes (*n*=882), of which 657 are higher in osteoblastoma and 225 are higher in osteosarcoma. Comparing osteoblastomas with MSCs/osteoblasts results in less differences (6%/7%) than with osteosarcomas (13%). This may imply that osteoblastomas are more similar to MSCs and osteoblasts than are osteosarcomas, thereby reflecting the difference in malignancy. The pathways that are most significantly altered when comparing osteosarcoma with osteoblastoma are the cell cycle, with an upregulation in malignant tumours, and pathways associated with cell division, especially regulation of the mitotic spindle. The significant pathways are listed in Table 3. To determine whether the larger size of the osteosarcoma group (*n*=25) underlies this difference in significant genes, we repeated the comparisons with only five osteosarcomas. Calculations were repeated 100 times for different combinations of 5 osteosarcomas and the results were averaged. The results are shown in Table 2, in the column labelled ‘avg of 100 × 5 OS’. This indeed resulted in a reduction in the number of significant genes, but the difference between osteosarcomas and MSCs or osteoblasts was still substantial, that is, 11% for MSCs and osteoblasts, whereas the comparison for osteoblastoma was only 6 or 7%.

## Discussion

Previous studies on genome-wide expression profiling of osteosarcoma have reported lists of genes that were found to be differentially expressed when comparing tumours with a poor histological response to chemotherapy and those with a good response (Ochi *et al*, 2004; Man *et al*, 2005; Mintz *et al*, 2005). Our study, comparing prechemotherapy biopsies from 8 good responders with those of 17 poor-responding patients did not result in a single significantly differentially expressed gene. Size and homogeneity of the patient cohort, type of expression profiling platform and statistical analysis may all account for this lack of significant genes. However, patient cohorts did not differ a lot in size, that is, 30, 28 and 13 cases, respectively, compared with 25 in our study, hence size seems to be a highly unlikely explanation for this difference. A long follow-up was available for our patient cohort for comparing the outcome of disease; however, this did not result in the identification of significantly differentially expressed genes.

Several meta-analysis studies on gene expression profiling provide a clarification for the lack of consistent results between different studies (Ein-Dor *et al*, 2005, 2006). reporting that there are many genes associated with different clinical behaviour, but the differences in expression are quite small and vary with different patient cohorts. They conclude that a significant set of genes for predicting survival requires thousands of patient samples. For a relatively rare tumour-like osteosarcoma, this is obviously not achievable, especially given the variation in clinical presentation and treatment of this tumour.

To identify the possible biological characteristics of osteosarcoma, a comparison of osteosarcoma expression profiles with profiles from their presumed progenitors, that is, MSCs and osteoblasts derived from these MSCs by *in vitro* differentiation, resulted in a large set of 2973 differentially expressed genes. This result validates our statistical analysis, thereby justifying the negative results obtained with the comparison within the osteosarcoma profiles. However, this set of genes is definitely contaminated with a subset that is the result of the different sources of primary tumour tissues and the *in vitro-*cultured MSCs and osteoblasts. Identification of common differentially expressed genes in osteosarcoma and benign osteoblastoma (most probably derived from the same progenitor cells, but with a completely different clinical behaviour) as compared with cultured MSCs and osteoblasts identified pathways that could most probably be attributed to the different sources of RNA. A subset of the 492 genes identified as commonly different in osteoblastoma and osteosarcoma when compared with cultured MSCs and osteoblasts could be assigned to specific pathways, thereby marking these as possible ‘culture-tissue artefacts’. Especially the most significant pathway identified by GenMAPP analysis, that is, upregulation of the MHC class II pathway in both osteosarcoma and osteoblastoma is the most obvious example, most probably caused by infiltrating cells that contaminate the tumour tissue, as has been described (Trieb *et al*, 1998).

Pathways characterised by an excess of differentially expressed genes between MSCs and osteosarcomas, but lacking the possible ‘culture-tissue artefacts’, are most likely involved in malignant transformation. The GO-Elite application (http://www.genmapp.org/go_elite/go_elite.html) generates a non-redundant list of significant signal transduction pathways from the Gene Ontology (GO) project from a gene list with specific criteria. The criteria in this study included genes with a significant difference in mRNA expression between osteosarcomas and MSCs or MSCs differentiated to osteoblasts. Criteria were strict and corrected for false discovery rate (FDR) due to multiple testing. On these restricted *P*-values, the GO-Elite algorithm imposes another FDR correction. Table 3 lists the pathways that survive this double FDR.

Pathways that subsist the FDR correction are involved in cell cycle regulation, mitosis and DNA replication, the usual suspects when comparing tumours with their progenitor cells. Osteosarcoma is especially characterised by a high growth rate and numerous mitotic figures (Kilpatrick and Renner, 2003) and chemotherapy protocols are aimed at inhibiting the cell cycle. However, the current protocols are not effective in 40% of cases (Lewis *et al*, 2007a) and this may be because of a variable expression of certain cell cycle components.

**Figure 7:**
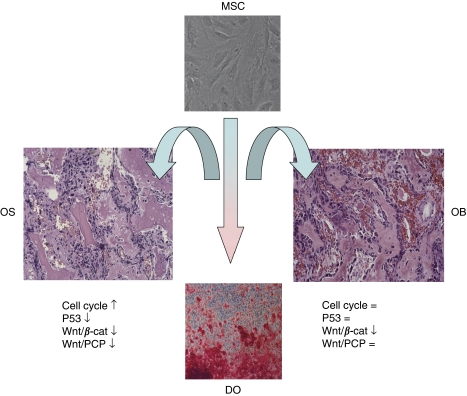
Proposed model for osteosarcomagenesis. Osteosarcomas and osteoblastomas originate from mesenchcymal stem cells that differentiate into osteoblasts. Increase in cell cycle activity and loss of Wnt/planar cell polarity signalling and P53 function contribute to malignancy.

Of special interest are developmental pathways that are known or suspected to have a role in osteosarcomagenesis. The Wnt signalling pathway shows downregulation when comparing MSCs or osteoblasts with osteosarcoma. Given the crucial role of this pathway in normal osteogenesis (Hartmann, 2006) and tumourigenesis in general, this observation suggests a role for Wnt signalling that differs from that in colorectal cancer, in which upregulation of the pathway is considered to be crucial for tumourigenesis (Klaus and Birchmeier, 2008). Indeed, we have recently shown with a functional reporter assay that Wnt/*β*-catenin signalling seems to be absent in osteosarcoma cell lines (Cai *et al*, 2009). In addition, we showed an absence of nuclear *β*-catenin staining in primary osteosarcomas, indicative of inactive Wnt/*β*-catenin signalling. Moreover, osteoblastomas showed a decrease in genes involved in Wnt/*β*-catenin signalling. The noncanonical WNT5A ligand, which is involved in Wnt/planar cell polarity (Qian *et al*, 2007), however, was not changed. Both observations in osteosarcoma and osteoblastoma can be clarified from the fact that Wnt/*β*-catenin signalling is important for maintaining cells in the MSC state (Baksh *et al*, 2007). Non-canonical Wnt signalling mediated by *WNT5A* antagonises this activity and promotes osteoblastogenesis of MSCs (Boland *et al*, 2004). Thus, loss of *WNT5A* expression may be a key event in malignant transformation in osteosarcoma. The findings of this study have led us to propose a model for osteosarcomagenesis, which is shown in Figure 7. An increase in Wnt signalling when comparing DO with MSCs is not observed. Wnt signalling changes during the process of differentiation and at different phases in osteoblastogenesis, whereby different Wnt activities are observed.

The comparison between osteoblastoma and the same presumed progenitor cells, MSCs and osteoblasts, did not result in pathways associated with cell cycle regulation. The profiles of osteoblastomas have fulfilled a dual purpose in this study: they were instrumental in identifying differentially expressed genes that resulted from a difference in cell culture and primary tissue, and they helped to recognise the cell cycle pathway as being most important for malignant transformation of osteosarcoma.

From this analysis it can be concluded that osteosarcoma differs from its presumed progenitor cells, MSCs and osteoblasts, in terms of cell cycle regulation and developmental pathways. Benign osteoblastomas with the same progenitor cells, but a much more favourable disease course, are not characterised by an increase in cell cycle but by a decrease in components of canonical Wnt signalling.

## Figures and Tables

**Table 1:** Clinical data

**Sample ID**	**Chip no.**	**Type**	**Age (years)**	**Gender**	**Subtype**	**Adj. CT**	**Chemo response**	**Overall survival**	**Metastasis**
L1370	IB10	Osteosarcoma	14	Male	HG Conv.	PIA	Good	Good	Lung
L1372	IB12	Osteosarcoma	10	Male	HG Conv.	AP	Good	Good	0
L1382	IB14	Osteosarcoma	16	Male	Tel.	PIA	Poor	Poor	Lung
L1385	IB16	Osteosarcoma	13	Female	Tel.	MA	Poor	Poor	Lung
L1016	IB19	Osteosarcoma	4	Male	HG Conv.	AP	Poor	Good	0
L2620	IB21	Osteosarcoma	16	Male	HG Conv.	AP	Poor	Poor	Lung+bone
L1375	IB22	Osteosarcoma	8	Male	HG Conv.	AP	Poor	Good	Local
L428	IB32	Osteosarcoma	16	Male	HG Conv.	AP	Poor	Good	Lung
L436	IB33	Osteosarcoma	18	Male	HG Conv.	MA	Poor	Good	0
L432	IB34	Osteosarcoma	17	Male	HG Conv.	AP	Poor	Poor	Lung
L361	IB35	Osteosarcoma	16	Female	HG Conv.	AP	Poor	Good	0
L1368	IB36	Osteosarcoma	10	Female	HG Conv.	PIA	Good	Good	0
L1376	IB37	Osteosarcoma	9	Female	HG Conv.	AP	Good	Good	0
L1386	IB38	Osteosarcoma	12	Female	HG Conv.	AP	Poor	Poor	Lung
L2702	IB39	Osteosarcoma	16	Male	HG Conv.	AP	Good	Poor	Lung
L2302	IB40	Osteosarcoma	19	Female	HG Conv.	AP	Poor	Good	0
L2296	IB41	Osteosarcoma	16	Male	HG Conv.	AP	Good	Poor	Lung+else
L2295	IB42	Osteosarcoma	40	Female	HG Conv.	AP	Poor	Good	0
L2611	IB43	Osteosarcoma	20	Female	HG Conv.	AP	Good	Good	0
L2300	IB44	Osteosarcoma	13	Male	HG Conv.	AP	Good	Good	0
L2294	IB45	Osteosarcoma	17	Female	HG Conv.	AP	Poor	Good	0
L2290	IB46	Osteosarcoma	36	Male	HG Conv.	AP	Poor	Poor	Local
L2301	IB47	Osteosarcoma	25	Male	HG Conv.	AP	Poor	Poor	Lung+else
L2281	IB48	Osteosarcoma	17	Male	HG Conv.	AP	Poor	Poor	Lung
L2289	IB54	Osteosarcoma	11	Male	HG Conv.	AP	Poor	Good	0
L578	IB55	Osteoblastoma	22	Male				Relapse	
L579	IB56^a^	Osteoblastoma	22	Male				Relapse	
L580	IB57	Osteoblastoma	13	Male				Remission	
L581	IB58	Osteoblastoma	16	Male				Remission	
L601	IB59	Osteoblastoma	44	Male				Remission	
									
FMSC-OB-diff	IB49	Osteoblasts							
MSC1-OB-diff	IB50	Osteoblasts							
220-OB-diff	IB51	Osteoblasts							
240-OB-diff	IB52	Osteoblasts							
MSC2-OB-diff	IB53	Osteoblasts							
MSC1	IB54	MSC							
MSC2	IB61	MSC							
C220R	IB62	MSC							
C240R	IB63	MSC							
FMSC	IB64	MSC							

Abbreviations: Adj. CT=adjuvant chemotherapy; AP=adriamycin and cisplatinum; HG=high grade; HG conv=high grade conventional; MA=methotrexate and adriamycin; MSC=mesenchymal stem cell; OB=osteoblastomas; PIA=cisplatinum, ifosfamide and adriamycin; Tel.=Telangiectatic.

a IB 56 is the recurrence from IB 55.

**Table 2:** Group comparisons and number of significant genes identified with Benjamini–Hochberg adjusted *P*-value

**Comparison**	**Total *P*<0.05**	**Up**	**Down**	**Avg of 100 × 5 OS**	**Presumed process**
OS *vs* MSC	2973	1159	1814	2456	Genes that are altered in osteosarcoma (OS) progression from MSC
OS *vs* DO	3041	1144	1897	2586	Genes that are altered in OS progression from differentiated osteoblasts (DO)
OS *vs* OB	882	225	657	937	Genes involved in malignancy of OS compared with benign osteoblastoma (OB)
DO *vs* MSC	369	175	194		Genes involved in MSC differentiation to osteoblasts
OB *vs* MSC	1245	606	639		Genes involved in osteoblastoma progression from MSC
OB *vs* DO	1573	770	803		Genes involved in osteoblastoma progression from osteoblasts

**Table 3:** Differentially expressed significant pathways

**Pathway**	***Z*-score**
*Comparison OS* *vs* *MSC*	
Macromolecule localization	5.99
Mitotic cell cycle checkpoint	5.00
DNA replication	4.57
Condensed chromosome, centromeric region	4.04
	
*Comparison OS* *vs* *DO*	
Negative regulation of S phase of mitotic cell cycle	5.34
	
*Comparison OS* *vs* *OB*	
Cell cycle	7.09
Spindle	6.34
IgG binding	5.69
Cell division	5.43
Condensed chromosome, centromeric region	5.36
Proteinaceous extracellular matrix	5.08
Chromosome segregation	4.94
DNA replication	4.80
	
*Comparison DO* *vs* *MSC*	
Cadmium ion binding	11.28
Trans-1,2-dihydrobenzene-1,2-diol dehydrogenase activity	7.39
Acute-phase response	5.57
Steroid biosynthetic process	5.14
Sterol metabolic process	5.12
Copper ion binding	4.45
Adipogenesis	4.67
	
*Comparison OB* *vs* *MSC*	
Developmental process	7.53
Cholesterol biosynthesis	7.36
Proteinaceous extracellular matrix	5.27
Cytokine and chemokine mediated signaling pathway	4.48
	
*Comparison DO* *vs* *OB*	
Negative regulation of transcription, DNA-dependent	5.58
Amine oxidase activity	4.99
Urogenital system development	4.94

Abbreviations: DO=differentiated osteoblasts; MSC=mesenchymal stem cell; OB=osteoblastoma; OS=osteosarcoma; *Z*-score=corrected score as determined by GO-elite. ,
