# Profiling of high-grade central osteosarcoma and its putative progenitor cells identifies tumourigenic pathways

**DOI:** 10.1038/sj.bjc.6605482

**Published:** 2009-12-08

**Authors:** A-M Cleton-Jansen, J K Anninga, I H Briaire-de Bruijn, S Romeo, J Oosting, R M Egeler, H Gelderblom, A H M Taminiau, P C W Hogendoorn

**Correction to**: *British Journal of Cancer* (2009) **101**, 1909–1918; doi:10.1038/sj.bjc.6605405

Upon publication of this paper in the last issue, an error was spotted in the clinical data in Table 1 – Chip number IB54 was incorrectly listed twice, when in the second instance it should have been referred to as IB60. The correct data are now shown below.

## Figures and Tables

**Table 1:** Clinical data

**Sample ID**	**Chip no.**	**Type**	**Age (years)**	**Gender**	**Subtype**	**Adj. CT**	**Chemo response**	**Overall survival**	**Metastasis**
L1370	IB10	Osteosarcoma	14	Male	HG conv.	PLA	Good	Good	Lung
L1372	IB12	Osteosarcoma	10	Male	HG conv.	AP	Good	Good	0
L1382	IB14	Osteosarcoma	16	Male	Tel.	PIA	Poor	Poor	Lung
L1385	IB16	Osteosarcoma	13	Female	Tel.	MA	Poor	Poor	Lung
L1016	IB19	Osteosarcoma	4	Male	HG conv.	AP	Poor	Good	0
L2620	IB21	Osteosarcoma	16	Male	HG conv.	AP	Poor	Poor	Lung+bone
L1375	IB22	Osteosarcoma	8	Male	HG conv.	AP	Poor	Good	Local
L428	IB32	Osteosarcoma	16	Male	HG conv.	AP	Poor	Good	Lung
L436	IB33	Osteosarcoma	18	Male	HG conv.	MA	Poor	Good	0
L432	IB34	Osteosarcoma	17	Male	HG conv.	AP	Poor	Poor	Lung
L361	IB35	Osteosarcoma	16	Female	HG conv.	AP	Poor	Good	0
L1368	IB36	Osteosarcoma	10	Female	HG conv.	PIA	Good	Good	0
L1376	IB37	Osteosarcoma	9	Female	HG conv.	AP	Good	Good	0
L1386	IB38	Osteosarcoma	12	Female	HG conv.	AP	Poor	Poor	Lung
L2702	IB39	Osteosarcoma	16	Male	HG conv.	AP	Good	Poor	Lung
L2302	IB40	Osteosarcoma	19	Female	HG conv.	AP	Poor	Good	0
L2296	IB41	Osteosarcoma	16	Male	HG conv.	AP	Good	Poor	Lung+else
L2295	IB42	Osteosarcoma	40	Female	HG conv.	AP	Poor	Good	0
L2611	IB43	Osteosarcoma	20	Female	HG conv.	AP	Good	Good	0
L2300	IB44	Osteosarcoma	13	Male	HG conv.	AP	Good	Good	0
L2294	IB45	Osteosarcoma	17	Female	HG conv.	AP	Poor	Good	0
L2290	IB46	Osteosarcoma	36	Male	HG conv.	AP	Poor	Poor	Local
L2301	IB47	Osteosarcoma	25	Male	HG conv.	AP	Poor	Poor	Lung+else
L2281	IB48	Osteosarcoma	17	Male	HG conv.	AP	Poor	Poor	Lung
L2289	IB54	Osteosarcoma	11	Male	HG conv.	AP	Poor	Good	0
L578	IB55	Osteoblastoma	22	Male				Relapse	
L579	IB56^a^	Osteoblastoma	22	Male				Relapse	
L580	IB57	Osteoblastoma	13	Male				Remission	
L581	IB58	Osteoblastoma	16	Male				Remission	
L601	IB59	Osteoblastoma	44	Male				Remission	
									
FMSC-OB-diff	IB49	Osteoblasts							
MSC1-OB-diff	IB50	Osteoblasts							
220-OB-diff	IB51	Osteoblasts							
240-OB-diff	IB52	Osteoblasts							
MSC2-OB-diff	IB53	Osteoblasts							
MSC1	IB60	MSC							
MSC2	IB61	MSC							
C220R	IB62	MSC							
C240R	IB63	MSC							
FMSC	IB64	MSC							

Abbreviations: Adj. CT=adjuvant chemotherapy; AP=adriamycin and cisplatinum; HG=high grade; HG conv.=high-grade conventional; MA=methotrexate and adriamycin; MSC=mesenchymal stem cell; OB=osteoblastoma; PIA=cisplatinum, ifosfamide and adriamycin; Tel.=telangiectatic.

a IB56 is the recurrence from IB55.

