# Supplementary material for: Profiling of high-grade central osteosarcoma and its putative progenitor cells identifies tumourigenic pathways
Source: Br J Cancer. 2009 Nov 3;101(11):1909–18. doi: 10.1038/sj.bjc.6605405 (PMC2788255; doi:10.1038/sj.bjc.6605405)
Supplement: Supplementary table 1 Legend [file 6605405x2.doc]

Legend Supplementary Table 1

OS: osteosarcoma, DO: differentiated osteoblasts, OB: osteoblastoma, MSC: mesenchymal stemcells, FC OS-DO: Fold-change difference in expression when comparing osteosarcoma and differentiated osteoblasts, pvalueadjOS – DO: adjusted p-value when comparing osteosarcoma and differentiated osteoblasts, culture-tissue genes: genes that are most probably differentially expressed as a result of comparing cultured cells with primary tissue.
